# Health-related quality of life and family functioning in parents of children with Barth syndrome: an application of the Double ABCX model

**DOI:** 10.1186/s13023-025-03636-0

**Published:** 2025-03-12

**Authors:** Yoonjeong Lim, Ickpyo Hong, Areum Han

**Affiliations:** 1 Division of Occupational Therapy, Decker College of Nursing and Health Sciences, Binghamton University, 4400 Vestal Parkway East, Binghamton, NY 13902 USA; 2 Department of Occupational Therapy, College of Software and Digital Healthcare Convergence, Yonsei University, 109 Backun Hall, Yonsei Univroad1, Wonju, 26493 Gangwon‑do South Korea; 3 Department of Occupational Therapy, University of Alabama at Birmingham, SHPB 339, 1720 2nd Ave South, Birmingham, AL 35294 USA

**Keywords:** Barth syndrome, BTHS, Family functioning, Functional performance, Health-related quality of life, And HRQoL

## Abstract

**Background:**

Living with children with disabilities has a significant impact on parental health-related quality of life (HRQoL) and family functioning. Barth syndrome (BTHS) is a rare, X-linked disorder that primarily affects males, presenting symptoms such as cardiomyopathy, neutropenia, muscle weakness, and growth delays. In this study, we investigated how a child’s functional performance, family cohesion, and satisfaction with healthcare affect parents of children with BTHS.

**Methods:**

Thirty-three parents of children with BTHS and 31 parents of age-matched unaffected children participated in this study. The parents completed a series of questionnaires. The Double ABCX model was applied to select measurement variables for this study. An independent samples *t*-test was used to compare HRQoL and family functioning between the two groups. Regression analysis was conducted to determine how a child’s functional performance, family cohesion, and satisfaction with healthcare affect HRQoL and family functioning of parents of children with BTHS.

**Results:**

The HRQoL and family functioning of parents of children with BTHS were significantly lower than those of unaffected children (*p* <.05). In the regression analysis, the child’s functional performance was a significant predictor of HRQoL and family functioning (*F*(3, 32) = 6.047, *p* =.003) for parents of children with BTHS.

**Conclusions:**

This study lays the groundwork for examining the impact of raising children with BTHS on parents and families. It is crucial for health professionals to understand the clinical features of BTHS and to consider not only the child but also the family in order to address their unmet needs and provide holistic healthcare services.

## Background

Barth syndrome (BTHS) is a rare X-linked genetic disorder that primarily affects males, resulting from a mutation in the TAFAZZIN gene (*TAZ*, G4.5) located on chromosome Xq28 [1]. Its primary symptoms include cardiomyopathy, neutropenia, muscle weakness, growth delays, and difficulty with physical exertion [1–2]. In the United States, BTHS occurs in approximately 1 in every 1,000,000 births [3]. BTHS causes problems in muscular and neurological function due to mitochondrial impairment and low levels of cardiolipin [4–5]. Mitochondria, the main energy producers in cells, require cardiolipin to maintain their structure and function; therefore, a deficiency in cardiolipin causes mitochondria to produce fewer of the essential lipids necessary for stability [6–7]. This leads to muscle weakness, limited exercise tolerance, and a quick onset of fatigue in individuals with BTHS [8]. As a result, strenuous activities can be particularly challenging for children with BTHS [8–9]. The existing literature extensively documented the genetic basis and natural history of BTHS [4–5, 8] and the physical symptoms and functional performance of individuals with BTHS [9–12]. However, there is currently no cure or targeted treatment for BTHS yet, which leads to parents and families of children with BTHS requiring continuous support to help manage the daily challenges they face, potentially impacting parental quality of life and family functioning.

Quality of life (QoL), often used interchangeably with health-related quality of life (HRQoL), is one of the key outcomes [13]. QoL reflects “an individual’s perception of their position in life in the context of the culture and value systems in which they live and in relation to their goals, expectations, standards, and concerns” [14]. HRQoL is a multidimensional construct that encompasses physical, emotional, cognitive, and social functioning, all of which contribute to a person’s overall health and well-being [15–16]. Several studies have shown that a child’s health condition affects parental HRQoL and family functioning [17–20]. Examining family impacts, such as parental HRQoL and family functioning, can help healthcare providers better understand the unmet needs of families managing challenges related to their child’s health condition [21]. However, investigating how a child’s illness, its progression, and its impact on the family interrelate is a complex and multifaceted process [22].

**Fig. 1:**
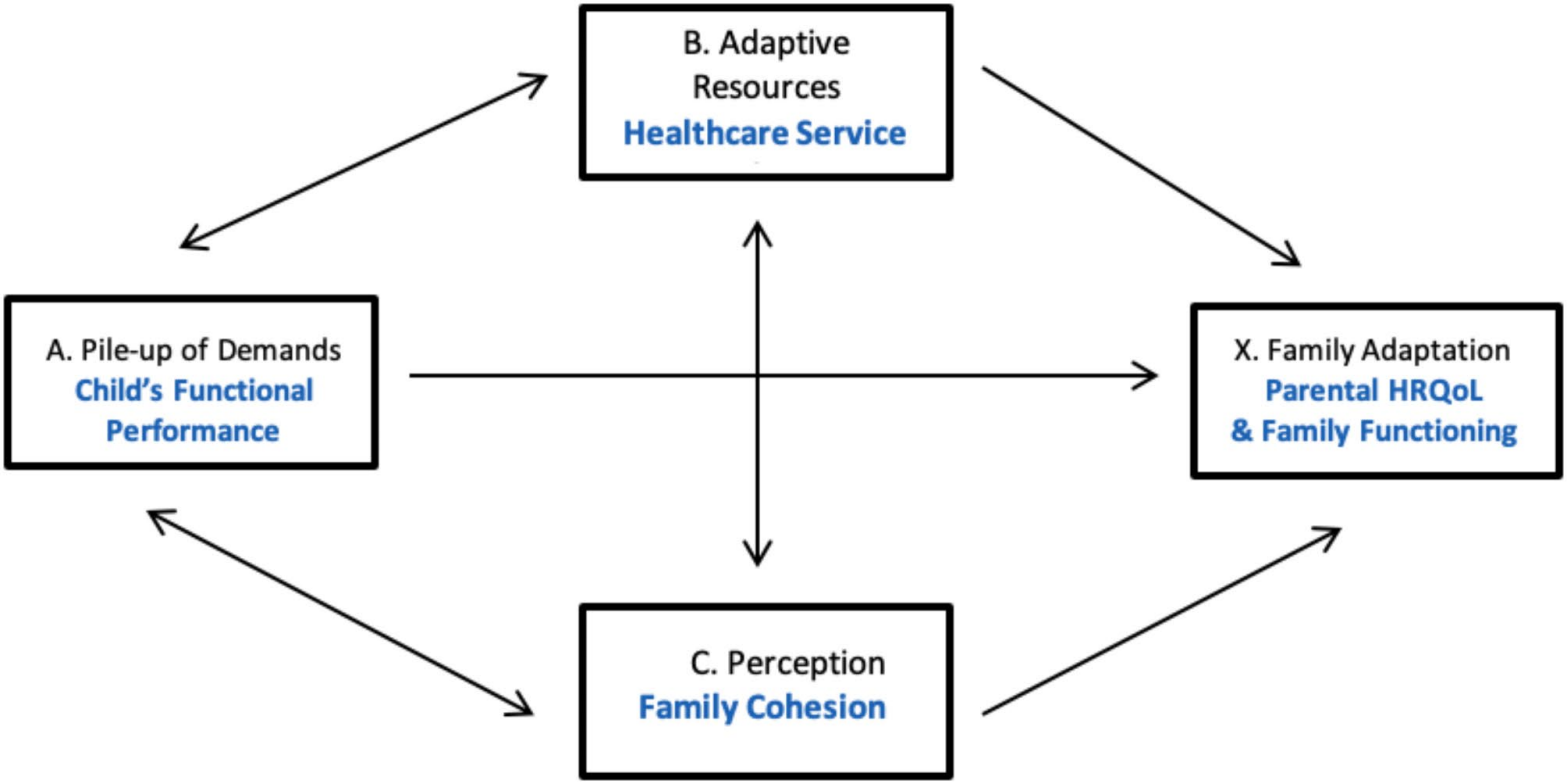
The double ABCX model

Researchers have sought to identify factors influencing the adaptation and adjustment processes of families with children with disabilities over time. The Double ABCX model [23] describes family recovery from a major event (e.g., a child’s disease) and the adaptation and adjustment processes of the families. The factors of this model include variables related to a child’s disease, family characteristics, and healthcare services. In Fig. 1, the A factor is the pile-up of demands (e.g., those associated with the child’s disease), and the B factor indicates adaptive resources, such as healthcare services. The C factor reflects the family’s perception of A, B, and the major event (e.g., the child’s disease). Adaptation (X) is the outcome of the family’s responses to these factors above. The Double ABCX model has been used in research exploring factors that influence families with children with disabilities [17, 24–26].

Currently, there is little evidence about the impact of raising children with BTHS on parents and families. Providing this evidence is crucial for understanding the daily challenges these parents and families encounter and for delivering services and support to address their unmet needs. Therefore, this study applied the Double ABCX model to examine how a child’s functional performance, family cohesion, and satisfaction with healthcare affect the parents of children with BTHS. Additionally, the health-related quality of life (HRQoL) and family functioning between parents of children with BTHS and those of unaffected children were compared in this study.

## Methods

### Participants

Thirty-three parents of children with BTHS and 31 parents of age-matched, unaffected children participated in this study. Eligibility criteria included: (1) being a parent of a child or youth diagnosed with BTHS or of an unaffected child between the ages of 4 and 19, and (2) speaking English and reading at an 8th-grade level. Parents of children with BTHS were recruited in collaboration with the Barth Syndrome Foundation, who attended the 7th and 8th Barth Syndrome Foundation International Scientific, Medical & Family Conferences. Parents of unaffected children were recruited through word of mouth, flyers posted in local areas, Facebook groups, and HealthStreet in Gainesville, FL. Both groups completed the PedsQL™ Family Impact Module and a demographic information form. Only parents of children with BTHS completed the Modified Barth Index, Family Adaptability and Cohesion Evaluation Scale-IV, and PedsQL™ Healthcare Satisfaction Generic Module. This study was approved by the University of Florida Institutional Review Board. Participating parents provided written informed consent.

### Instruments

#### The Pedsql™ Family Impact Module

The PedsQL™ Family Impact Module (PedsQL™ FIM) assesses the impact of pediatric chronic health conditions on parental HRQoL and family functioning [27]. This self-report questionnaire consists of 8 subscales with 36 items: (1) Physical Functioning, (2) Emotional Functioning, (3) Social Functioning, (4) Cognitive Functioning, (5) Communication, (6) Worry, (7) Daily Activities, and (8) Family Relationships. Each item is scored on a 5-point scale (never a problem = 0, almost never = 1, sometimes = 2, often = 3, almost always = 4) and transformed to a 0 to 100 scale (never a problem = 100, almost never = 75, sometimes = 50, often = 25, almost always = 0). The total score of the PedsQL™ Family Impact Module is calculated by averaging the 36 items. Within this module, the Parental HRQoL score is calculated by averaging items from the Physical Functioning, Emotional Functioning, Social Functioning, and Cognitive Functioning subscales, while the Family Functioning score is derived from the Daily Activities and Family Relationships subscales. Higher scores indicate better parental HRQoL and family functioning.

#### The Modified Barthel Index

The Modified Barthel Index (MBI) is a 10-item questionnaire that measures a child’s performance in activities of daily living (i.e., the child’s functional ability) [28]. The MBI consists of 10 subscales: (1) Personal Hygiene, (2) Bathing Self, (3) Feeding, (4) Toileting, (5) Stair Climbing, (6) Dressing, (7) Bowel Control, (8) Bladder Control, (9) Ambulation/Wheelchair, and (10) Chair/Bed Transfers. The MBI is administered by a therapist or family member/friend based on their observations of the child’s daily life. Each item is scored from “unable to perform task” to “fully independent” (0–5 for Personal Hygiene and Bathing Self; 0–10 for Feeding, Toilet Transfer, Dressing, Bladder Control, Bowel Control, and Stair Climbing; 0–15 for Chair/Bed Transfer and Ambulation/Wheelchair). The total score is calculated by summing the 10 items and ranges from 0 to 100. Higher scores indicate greater functional ability or independence in activities of daily living.

#### The Family Adaptability and Cohesion Evaluation Scale-IV

The Family Adaptability and Cohesion Evaluation Scale-IV (FACES-IV) is a 42-item questionnaire that measures the dimensions of family cohesion and family flexibility [29]. It consists of 6 subscales: (1) Balanced Cohesion, (2) Balanced Flexibility, (3) Disengaged, (4) Enmeshed, (5) Rigid, (6) Chaotic. Each item is rated on a 5-point scale (strongly disagree = 1, generally disagree = 2, undecided = 3, generally agree = 4, strongly agree = 5). Raw subscale scores are converted to percentiles using a percentile conversion chart. The total Circumplex Ratio is computed from the 6 subscales as follows, with values above 1 indicating a more balanced/healthy family dynamic.

Cohesion Ratio = Balanced Cohesion / ((Disengaged + Enmeshment) / 2).

Flexibility Ratio = Balanced Flexibility / ((Rigid + Chaotic) / 2).

Total Circumplex Ratio = (Cohesion Ratio + Flexibility Ratio) / 2.

#### The Pedsql™ Healthcare Satisfaction Generic Module

The PedsQL™ Healthcare Satisfaction Generic Module (PedsQL™ HS) measures healthcare satisfaction [30]. It consists of 6 subscales with 27 items: (1) Information (5 items), (2) Inclusion of Family (4 items), (3) Communication (5 items), (4) Technical Skills (3 items), (5) Emotional Needs (4 items), and (6) Overall Satisfaction (3 items). Each item is scored on a 5-point scale (never = 100, sometimes = 75, often = 50, always = 25, not applicable = 0). The total PedsQL™ HS score is calculated by averaging the six subscales. Higher scores indicate greater satisfaction with healthcare.

#### The demographic information form

The demographic information form, generated by the investigators of this study, consisted of (1) the child’s date of birth and age, (2) the parent’s age, (3) marital status, (4) race, (5) employment, (6) level of education, and (7) family income.

### Data analysis

The Shapiro-Wilk was used to test the normality of the data. For child and parent demographic variables, an independent samples *t*-test was used for continuous variables (e.g., child’s age), and the Chi-square test was used for categorical variables (e.g., parent education). The PedsQL™ FIM scores were compared between the parents of children with BTHS and parents of unaffected children using an independent samples *t*-test and an adjusted method. In the adjusted method, propensity score matching with inverse probability of treatment weighting (PS-IPTW) [31–32] was applied to control for demographic differences between the two groups when comparing the mean scores of the PedsQL™ FIM. The correlation between parental HRQoL and family functioning (PedsQL™ FIM) with a child’s functional performance (MBI), family cohesion (FACES-IV), and satisfaction with healthcare (PedsQL™ HS) was analyzed using the Spearman correlation coefficient. Regression analysis was conducted to examine variables that significantly predict parental HRQoL and family functioning in the parents of children with BTHS. The independent variables were the child’s functional performance (MBI), family cohesion (FACES-IV), and satisfaction with healthcare (PedsQL™ HS). The dependent variable was parental HRQoL and family functioning (PedsQL™ FIM). Statistical analyses were performed using SAS statistical software version 9.4 (SAS Institute, Inc.). The alpha level was set at 0.05.

## Results

### Summary statistics for child and parent demographic variables

Sixty-four parents participated in this study, including 33 parents of children with BTHS and 31 parents of unaffected children. Table 1 displays summary statistics for child and parent demographic variables. Parent race (*p* =.0002) and parent education (*p* =.0002) were significantly different between the two groups. Parent race differed significantly, with 30 (90.91%) parents being non-Hispanic White in the BTHS group compared to 15 (48.39%) in the unaffected group. In addition, 23 (74.19%) parents in the unaffected group had a graduate or professional degree, compared to 9 (27.27%) in the BTHS group.

**Table 1:** Summary statistics for child and parent demographic variables

Characteristics	BTHS (*n* = 33)	Unaffected (*n* = 31)	*p*-value
**Child Age** Age range Median	11.53 (4.65) 4–19 11.75	11.44 (3.94) 5–19 11.11	0.934
**Child Sex**			
Male	33	31	
**Parent Age** Age range Median	42.45 (6.97) 28–54 44	42.90 (6.60) 30–54 44	0.793
**Parent Race**			0.0002
Non-Hispanic white	30 (90.91)	15 (48.39)	
Other	3 (9.09)	16 (51.61)	
**Parent Education**			0.0002
Graduate or professional degree	9 (27.27)	23 (74.19)	
Less than graduate or professional degree	24 (72.73)	8 (25.81)	
**Marital Status**			0.9247
Married	29 (87.88)	27 (87.10)	
Other	4 (12.12)	4 (12.90)	
**Employment Status**			0.1016
Full-time	18 (54.55)	23 (74.19)	
Less than full-time	15 (45.45)	8 (25.81)	
**Family Income**			0.9938
$80,000 or above	16 (48.48)	15 (48.39)	
Less than $80,0000	17 (51.52)	16 (51.61)	

Note. Mean (standard deviation) for child age; *n* (%) for the others (e.g., child sex, parent race, etc.); categorical variables were analyzed by Chi-square test. Continuous variables were analyzed by independent *t*-test

### Parental HRQoL and family functioning between the two groups

In the PedsQL™ FIM, all subscale scores, parental HRQoL, family functioning, and the total score between the parents of children with BTHS and parents of unaffected children were significantly different (Table 2). The Worry subscale demonstrated the highest mean difference between the two groups, followed by the Communication subscale. After controlling for the significantly different demographic variables (parent race and education) using the PS-IPTW adjusted method, the Physical Functioning and Cognitive Functioning subscales were not significantly different between the two groups.

**Table 2:** Mean score differences of the pedsql™ family impact module between the unaffected and BTHS groups

	Unadjusted^a^					PS-IPTW adjustment^b^	
	BTHS (*n* = 33)	Unaffected (*n* = 31)	Mean difference (SE)		*p*	Mean difference (SE)	*p*
**Parental HRQoL**	61.40 (21.59)	76.23 (15.14)	14.82 (4.68)	0.002		12.92 (4.40)	0.005
Physical Functioning	60.61 (26.29)	75.27 (19.36)	14.66 (5.80)	0.014		9.70 (4.98)	0.057
Emotional Functioning	59.39 (21.50)	73.87 (18.65)	14.47 (5.04)	0.005		15.93 (5.43)	0.005
Social Functioning	61.10 (27.12)	78.02 (19.76)	16.92 (5.96)	0.006		16.07 (5.79)	0.007
Cognitive Functioning	63.79 (25.28)	77.74 (16.42)	13.95 (5.29)	0.011		10.27 (5.17)	0.052
**Communication**	59.09 (17.23)	88.17 (12.69)	29.08 (3.80)	< 0.001		27.49 (4.05)	< 0.001
**Worry**	47.73 (19.33)	86.13 (21.16)	38.40 (5.06)	< 0.001		42.41 (4.19)	< 0.001
**Family Functioning**	57.23 (21.46)	73.79 (19.46)	16.56 (5.10)	0.001		16.58 (5.28)	0.002
Daily Activities	49.75 (31.56)	71.77 (21.70)	22.02 (6.73)	0.002		23.62 (6.87)	0.001
Family Relationships	62.54 (20.72)	75.81 (19.28)	13.26 (5.01)	0.01		11.23 (5.24)	0.037
**Total Score**	58.37 (18.03)	78.35 (14.75)	19.97 (4.13)	< 0.001		19.46 (3.92)	< 0.001

Note. *M* (*SD*) for unaffected and BTHS; propensity score matching with inverse probability of treatment weighting (PS-IPTW); standard error (*SE*)

^a^ Independent-samples *t*-test between the unaffected and BTHS groups

^b^ After applying propensity score matching with inverse probability of treatment weighting, all demographics were balanced between the unaffected and BTHS groups

### Correlation analysis in the BTHS groups

The MBI and PedsQL™ HS did not meet the normality test (*p* <.05); therefore, the Spearman correlation coefficient, a non-parametric approach, was used in this study (Table 3). In the correlation analysis, the child’s functional performance showed a positive moderate correlation with parental HRQoL (*r*(33) = 0.561, *p* <.001) and the total score (*r*(33) = 0.547, *p* <.001). Family cohesion demonstrated a positive moderate relationship with family functioning (*r*(33) = 0.414, *p* =.016).

**Table 3:** Correlation analysis in the BTHS group

	PedsQL™ Family Impact Module		
	Parental HRQoL	Family Functioning	Total Score
**MBI**	0.561^**^	0.342	0.547^**^
**FACES-IV**	0.303	0.414^*^	0.296
**PedsQL™ Healthcare Satisfaction**	0.175	0.314	0.264

^*^p <.05

^**^p <.01

### Regression analysis in the BTHS group

Multiple regression analysis was conducted to determine the predictors of parental HRQoL and family functioning in parents of children with BTHS (Table 4). In this model, 38.5% of parental HRQoL and family functioning in parents of children with BTHS was significantly explained by the three independent variables (*F*(3, 32) = 6.047, *p* =.003). The child’s functional performance factor significantly predicted parental HRQoL and family functioning in the parents of children with BTHS (*p* =.0008). However, family cohesion and satisfaction with healthcare variables were not significant predictors in this model.

**Table 4:** Regression analysis in the BTHS group

Variable	Parental HRQoL and family functioning		
	*B*	*SE*	*p*
Child’s Functional Ability	0.586	0.167	0.0008^*^
Family cohesion	2.745	2.809	0.1589
Satisfaction with healthcare	0.157	0.145	0.8028
*R* ^*2*^	0.385		
*F*	6.047^*^		

*SE* = standard error

^*^p <.05

## Discussion

By applying the Double ABCX model, this study investigated the impact of a child’s functional performance, family cohesion, and satisfaction with healthcare on parents of children with BTHS. The HRQoL and family functioning of parents of children with BTHS were compared to those of parents of unaffected children. Parents of children with BTHS demonstrated significantly lower HRQoL and family functioning than those of unaffected children, which is consistent with previous studies conducted with other clinical populations [17–18, 21, 33–34]. Among the PedsQL™ FIM scales, the Worry and Communication subscales showed the highest mean differences between the two groups. These differences reflect worries about their child’s future and the impact of others on the child’s condition, as well as feelings that others do not understand the family’s situation and difficulties talking to others about their child’s health.

According to the correlation and regression analyses, the child’s functional performance was a significant factor affecting parental HRQoL and family functioning of parents of children with BTHS. This finding is consistent with previous studies that the severity of a child’s function significantly predicted parental HRQoL [17, 35]. This is not unexpected, as BTHS represents muscle weakness, which could affect overall physical function. Due to their physical symptoms, children with BTHS face challenges in participating in strenuous physical activities [8, 9]. These physical symptoms may restrict their participation in important and meaningful age-related activities, as well as in everyday activities across home, school, and community settings. Therefore, an in-depth exploration of the level of participation in everyday activities for children with BTHS and its impact on their families is crucial.

Olson [29] noted that most FACES-IV scores, which measure family cohesion, typically range from 0 to 2. However, in this study, the majority of parents of children with BTHS scored higher than 2 on the FACES-IV. This indicates that these parents maintained a high level of family cohesion, resulting in less variability in the data for the correlation and regression analyses. Since the parents in this study had high levels of education and middle to upper-middle-class incomes, this might contribute to their heightened capacity to cope with their child’s diseases [36]. It is reported that raising children with disabilities may strengthen parents’ inner resilience and improve family cohesion [37]. By gaining a deeper understanding of their child’s disability, parents may experience positive personal growth and improved relationships [38]. Therefore, the findings from this study may indicate that parents of children with BTHS have adjusted to managing their child’s disease conditions.

For families providing lifelong care for their children, the caregiving burden often increases as the disease progresses [39]. As such, ongoing disease management support, updated information, and education are essential. Additionally, families may benefit from counseling to improve their adaptive skills for managing the growing burden and responsibilities [18]. Health professionals should consider offering family education, coaching on caregiving techniques and strategies, encouraging participation in activities outside the home, and connecting them with others caring for children with disabilities for additional support [40–41]. Most importantly, to offer the most effective services to families of children with rare diseases, health professionals must understand both the symptoms of pediatric rare diseases and the experiences and unmet needs of the families, ensuring timely and necessary support.

This study has several limitations. Participants were recruited through the Barth Syndrome Foundation (BSF), which could potentially result in a selection bias. The BSF offers parent support groups and valuable up-to-date resources, which are crucially important to patient care and parental advocacy. Parents who were more engaged with the BSF may be more resilient and better prepared to handle caregiving demands, creating a sample that may not fully reflect all parents of children with BTHS. Additionally, most participants were non-Hispanic White, with middle to upper-middle-class incomes and high education levels, restricting the diversity of perspectives and experiences. Families from various racial, ethnic, and socioeconomic backgrounds may have unique caregiving experiences, which could affect their HRQoL and family functioning.

Future research should aim to include a more diverse sample representing a broader range of income, education, socioeconomic status, and racial/ethnic groups to ensure that findings are applicable to a broader population of parents of children with BTHS. Moreover, it is important to consider a longitudinal approach to examine caregiving impacts on the family over time, such as challenges as the child ages or the disease progresses. Lastly, future research might consider incorporating self-reported data with objective measures or multiple informants (e.g., healthcare providers) to provide a more comprehensive view of the caregiving experience.

## Conclusion

The parents of children with BTHS reported lower HRQoL and family functioning compared to those of unaffected children, and a child’s functional performance was a significant factor affecting parents and families. The findings of this study lay the groundwork for exploring the impact of raising children with BTHS on parental HRQoL and family functioning. It is crucial for health professionals to understand the clinical features of BTHS and to consider not only the child but also the family in order to address their unmet needs and provide holistic healthcare services. They should implement practical strategies to support positive family functioning and well-being in daily life.

## Data Availability

The authors confirm that all data generated or analyzed during this study. The datasets used for this study are available from the corresponding author upon reasonable request.

## Abbreviations

BTHS: Barth Syndrome
HRQoL: Health-related quality of life
QoL: Quality of life

## References

[1] Taylor C, et al. Clinical presentation and natural history of Barth syndrome: an overview. J Inherit Metab Dis. 2022;45(1):7–16. 34355402 10.1002/jimd.12422

[2] Barth PG, et al. An X-linked mitochondrial disease affecting cardiac muscle, skeletal muscle and neutrophil leucocytes. J Neurol Sci. 1983;62:327–55. 6142097 10.1016/0022-510x(83)90209-5

[3] Miller PC, et al. A bayesian analysis to determine the prevalence of Barth syndrome in the pediatric population. J Pediatr. 2020;217:139–44. 31732128 10.1016/j.jpeds.2019.09.074

[4] Spencer CT, et al. Cardiac and clinical phenotype in Barth syndrome. Pediatrics. 2006;118:337–46. 10.1542/peds.2005-2667 16847078

[5] Barth PG, et al. X-linked cardioskeletal myopathy and neutropenia (Barth syndrome): an update. Am J Med Genet A. 2004;126a:349–54. 10.1002/ajmg.a.20660 15098233

[6] Schlame M, et al. Deficiency of tetralinoleoyl-cardiolipin in Barth syndrome. Ann Neurol. 2002;51:634–7. 12112112 10.1002/ana.10176

[7] Ren M, Phoon CK, Schlame M. Metabolism and function of mitochondrial Cardiolipin. Prog Lipid Res. 2014;55:1–16. 24769127 10.1016/j.plipres.2014.04.001

[8] Clarke SL, et al. Barth syndrome. Orphanet J Rare Dis. 2013;8:23. 23398819 10.1186/1750-1172-8-23 PMC3583704

[9] Hornby B et al. Functional exercise capacity, strength, balance and motion reaction time in Barth syndrome. Orphanet J Rare Dis. 2019;14. 10.1186/s13023-019-1006-8 PMC6371525 30744648

[10] Bittel AJ, et al. Reduced muscle strength in Barth syndrome May be improved by resistance exercise training: A pilot study. JIMD Rep. 2018;41:63–72. 10.1007/8904_2018_10. 29654548 10.1007/8904_2018_102 PMC6122057

[11] Chu VW, Reynolds S, Payne S, Hunter M et al. The relationship between participant-reported energy levels & energy expenditure & sleep among individuals with Barth syndrome. The American Journal of Occupational Therapy. 2023;77(Supplement_2). 7711500050p1-7711500050p1.

[12] Reynolds S, Babson I, Daw E. (2023). A Qualitative investigation of fatigue & its daily impacts as perceived by individuals with Barth syndrome & their families. The American Journal of Occupational Therapy. 2023;77(Supplement_2). 7711510338p1-7711510338p1.

[13] American Occupational Therapy Association. Occupational therapy practice framework: domain and Process—Fourth edition. Am J Occup Ther. 2020;74(Supplement2):p74124100101–74. 10.5014/ajot.2020.74S2001 34780625

[14] WHOQOL Group. Study protocol for the world health organization project to develop a quality of life assessment instrument (WHOQOL). Qual Life Res. 1993;2:153–9. 8518769

[15] Hays RD, et al. Agreement between self reports and proxy reports of quality of life in epilepsy patients. Qual Life Res. 1995;4:159–68. 7780382 10.1007/BF01833609

[16] Kaplan RM. Quality of life measurement. In: Karoly P, editor. Measurement strategies in health psychology. John Wiley; 1985.

[17] Lim Y. Impact of Raising children with rare diseases on parental quality of life and family functioning. Int J Rare Dis Disord. 2023;6:1–6.

[18] Ammann-Schnell L, et al. The impact of severe rare chronic neurological disease in childhood on the quality of life of families-a study on MLD and PCH2. Orphanet J Rare Dis. 2021;16:211. 33971942 10.1186/s13023-021-01828-y PMC8111977

[19] Hsieh RL, et al. Quality of life, health satisfaction and family impact on caregivers of children with developmental delays. Child Care Health Dev. 2009;35:243–9. 19134010 10.1111/j.1365-2214.2008.00927.x

[20] Limbers CA, et al. A comparative analysis of health-related quality of life and family impact between children with ADHD treated in a general pediatric clinic and a psychiatric clinic utilizing the PedsQL. J Atten Disord. 2011;15:392–402. 20065072 10.1177/1087054709356191

[21] Lim Y, Hong I, Han A. The impact of Raising children with Barth syndrome on parental health-related quality of life and family functioning: preliminary reliability and validity of the pedsql™ family impact module. Occup Ther Int.2023:1–6. 10.1155/2023/5588935 PMC10771332 38187035

[22] Panepinto JA, Hoffmann RG, Pajewski NM. A psychometric evaluation of the PedsQL family impact module in parents of children with sickle cell disease. Health Qual Life Outcomes. 2009;7:32. 19371442 10.1186/1477-7525-7-32 PMC2678996

[23] McCubbin HI, Patterson JM. The family stress process: the double ABCX model of adjustment and adaptation. Marriage Family Rev. 1983;6:7–37.

[24] Pozo P, Sarriá E, Brioso A. Family quality of life and psychological well-being in parents of children with autism spectrum disorders: A double ABCX model. J Intellect Disabil Res. 2014;58:442–58. 23600450 10.1111/jir.12042

[25] Frishman N et al. Perceived quality of life among caregivers of children with a childhood-onset dystrophinopathy: A double ABCX model of caregiver stressors and perceived resources. Health Qual Life Outcomes. 2017;15. 10.1186/s12955-017-0612-1 PMC5303295 28187773

[26] Boettcher J et al. Perceived mental health in parents of children with rare congenital surgical diseases: A double ABCX model considering gender. Orphanet J Rare Dis. 2021;16. 10.1186/s13023-021-01998-9 PMC8427164 34503547

[27] Varni JW, et al. The PedsQL family impact module: preliminary reliability and validity. Health Qual Life Outcomes. 2004;2:55. 15450120 10.1186/1477-7525-2-55 PMC521692

[28] Shah S, Vanclay F, Cooper B. Improving the sensitivity of the Barthel index for stroke rehabilitation. J Clin Epidemiol. 1989;42:703–9. 2760661 10.1016/0895-4356(89)90065-6

[29] Olson DH. The Family Adaptability and Cohesion Evaluation Scale-IV (FACES-IV) manual. Life Innovations;2010.

[30] Varni JW, Seid M, Kurtin PS. PedsQL 4.0: reliability and validity of the pediatric quality of life inventory version 4.0 generic core scales in healthy and patient populations. Med Care. 2001;39:800–12. 11468499 10.1097/00005650-200108000-00006

[31] Rosenbaum PR, Rubin DB. Assessing sensitivity to an unobserved binary covariate in an observational study with binary outcome. Kournal Royal Stat Society: Ser B (Methodological). 1983;45:212–8.

[32] Rosenbaum PR, Rubin DB. Reducing bias in observational studies using subclassification on the propensity score. J Am Stat Assoc. 1984;79:516–24.

[33] Brehaut JC, et al. The health of primary caregivers of children with cerebral palsy: how does it compare with that of other Canadian caregivers? Pediatrics. 2004;114:e182–91. 15286255 10.1542/peds.114.2.e182

[34] Lim Y, et al. Preliminary reliability and validity of the PedsQL^™^ family impact module in parents of children with congenital muscular dystrophy. J Rare Dis. 2024;3. 10.1007/s44162-024-00057-8.

[35] Wang M, et al. Severity of disability and income as predictors of parents’ satisfaction with their family quality of life during early childhood years. Res Pract Persons Severe Disabl. 2004;29:82–94.

[36] Patterson JM. Understanding family resilience. J Clin Psychol. 2002;58:233–46. 11836706 10.1002/jclp.10019

[37] Reichman NE, Corman H, Noonan H. Impact of child disability on the family. Matern Child Health J. 2008;12:679–83. 18060488 10.1007/s10995-007-0307-z PMC11242919

[38] Pakenham KI, Sofronoff K, Samios C. Finding meaning in parenting a child with asperger syndrome: correlates of sense making and benefit finding. Res Dev Disabil. 2004;25:245–64. 15134791 10.1016/j.ridd.2003.06.003

[39] Bray P, et al. Health status of boys with Duchenne muscular dystrophy: A parent’s perspective. J Paediatr Child Health. 2011;47:557–62. 21392149 10.1111/j.1440-1754.2011.02022.x

[40] Angelini C, Tasca E. Fatigue in muscular dystrophies. Neuromuscul Disord. 2012;22(3–3):S214–20. 23182642 10.1016/j.nmd.2012.10.010 PMC3526799

[41] Storch EA, et al. Psychosocial functioning in youth with Barth syndrome. Child Health Care. 2009;38:137–56. 20808735 10.1080/02739610902813344 PMC2929969

